# Stimulants in the Treament of Disease

**Published:** 1870-10

**Authors:** 


### Editorial Department.

### Stimulants in the Treament of Disease.

It is well known by the profession that not until within the last fifteen years was it common for physicians to prescribe stimulants in disease; it appears to have succeeded to the much more objectionable plan of depletion, low diet, sedatives, etc., so prevalent with the medical fathers of the present time in their earlier days. When it had become quite apparent that most patients would recover sooner and more fully if the depletive measures were omitted, and generous alimentation substituted therefor, the second step, very easy and quite natural, was soon taken, and stimulants added, soon coming to be regarded as indispensable in some stages of nearly all forms of disease. Early in the history of the introduction of stiumlants as a therapeutic measure, it was announced that whisky was a sovereign remedy for consumption. Unfortunately, for the early use of the remedy, it was said, that the cheapest, strongest-smelling, and most disagreeable, unrectified article, the "Irish whisky of forty rods," was the most useful in the treatment of this disease, and this operated to check for a time its use. But the "Best Rye" and "Kentucky Bourbon" early found advocates; when, by a general consent, all sick people commenced its use, and nearly all well people followed the example, believing it preventive as well as curative. From a very legitimate use of stimulus in conditions of great temporary depression, physicians extended the range of its usefulness indefinitely, and gave the sanction of their advice to the adoption of a measure when, as yet, there was no adequate evidence of its usefulness. It grew in favor with the profession and with the public from an inherent property, which has always lent to it an irresistible charm; and, sustained by professional approbation and popular favor it come to be the one all-controlling, everywhere-present, almighty "spirit" of good;—capable, of course, of some evil if misapplied, but in its proper use the universal restorer;—good for drink, for food, and for medicine.

At length, after irreparable injury, a few of the very observing men of the profession hesitate as regards its benign influence, and slowly and reluctantly admit the possibility of a mistake. The doubt being raised, the question is now fully open for discussion: Is Alcohol useful in the treatment of disease; and, if so, in what forms and to what extent?

We do not propose to definitely settle the question, but simply announce that it is open for consideration, believing that it will be eventually presented in all its bearings and that a more definite and truthful idea will be gained of the influence, usefulness and injury of stimuli in conditions of disease. For the last ten years, whisky has been the staple of treatment in consumption, and this is a period sufficient to afford some results indicating its true worth. It has been combined with Cod Liver Oil and with various inert compounds, and its effects alone have not been so much observed. Yet we cannot doubt that experience in the use of stimulants, as curative of disease, has already been quite adequate to the formation of some positive conclusions. Great differences of opinion are to be expected, and probably the profession cannot agree in the effects of this, any more than of other medicinal substances; still, we think it may be said, in all fairness, that we are not *certain* that anything has been gained. We have ourselves never observed carefully a single case, taken during the entire period of the disease, which we could think in the end benefitted; while many, very many, have been compelled to abandon it altogether or to restrict its use to inappreciable quantities. The most common ill-effects observed is diminished relish and desire for food—which appears to be the usual and almost uniform effects—an excited circulation, and an irritable condition of the nervous system. The habit of prescribing stimulus for invalids having symptoms of defective nutrition is almost universal, and is based mainly upon the general idea that Alcohol increases nutrition or is itself nutritious. Dr. Flint, in his Physiology, says: "That it may temporarily give tone and vigor to the system, when the energies are unusually taxed, cannot be doubted; but this effect is not produced in all individuals. The constant use of Alcohol may create an apparent necessity for it, producing a condition of the system which must be regarded as pathological." Physiologists have thus far shown, so far as they have shown anything, that it is not an aliment, and is not essential to the highest state of nutrition. Dr. Hammond has made some very interesting experiments, going to show its effects upon nutrition; and if he has shown that it is increased by it, he has shown also that at the same time the health was impaired. After taking sixty drachms of Alcohol in five days, his weight had increased a little; he says: "during this time there was some disturbance of the general health. The pulse was increased in frequency; there was headache, and the mental faculties were not so clear as on the days when no Alcohol was taken." But we have no time to answer the question that we ask. We leave the answer to the observation of the profession. Stimulus in disease, so far as our own observation extends, should be confined to conditions of temporary exhaustion, and cannot be relied upon as an efficient agent of repair. It is the fashionable remedy in consumption, and has thus been extended to all supposed—as well as real—cases of this disease. Some instances might doubtless be cited where at least it did no harm, but we have no doubt that it has done infinite injury, taking its use in the aggregate, partly perhaps from its abuse rather than its legitimate use. We have been led to these remarks mainly for the purpose of calling attention to this very important therapeutic agent, and possibly being thus able to collect the opinions of those whose opportunities of observing its effects have been sufficient to determine its value.

